# Zoonotic threat of cystic echinococcosis in Tunisia: insights into livestock prevalence and identification of the G1 genotype

**DOI:** 10.3389/fvets.2025.1536368

**Published:** 2025-02-20

**Authors:** Mohamed Hedi Abdelghani, Selim M’rad, Raja Chaâbane-Banaoues, Sayadi Taoufik, Mohamed Ali Charfedine, Lamia Zemzemi, Ines Kamoun, Hamouda Babba, Myriam Oudni-M’rad

**Affiliations:** ^1^Laboratory of Medical and Molecular Parasitology-Mycology (LP3M), LR12ES08, Faculty of Pharmacy, University of Monastir, Monastir, Tunisia; ^2^Central Slaughterhouse of Sousse, Sousse, Tunisia; ^3^Slaughterhouse of Ksour Essef, Mahdia, Tunisia; ^4^Maternity and Neonatology Center, University Hospital F. Bourguiba, Monastir, Tunisia

**Keywords:** cystic echinococcosis, *Echinococcus granulosus*, hydatidosis, livestock, prevalence, fertility, viability, genotype

## Abstract

**Introduction:**

Cystic echinococcosis (CE) is a zoonosis caused by the larval stage of the *Echinococcus granulosus sensu lato* (*s.l*.) complex. CE is globally distributed, with a particularly high prevalence in North African countries, especially Tunisia. Despite its significant public health impact and the economic burden it places on livestock production, recent data on CE prevalence in Tunisian livestock remain scarce. This study aimed to assess the prevalence of CE in livestock, investigate potential differences across host species, and identify risk factors contributing to the CE transmission dynamics.

**Methods:**

The study was conducted in two governorates located in the North-Eastern region of Tunisia. A multidimensional approach included post-mortem inspection of slaughtered animals, fertility and viability analyses of the isolated CE cysts, and molecular genotyping of the parasite was conducted.

**Results and discussion:**

A total of 21,487 animals were examined, 15.86% of the sheep and 9.57% of the cattle were infected with at least one CE cyst, with females showing higher prevalence rates. No CE cases were detected in goats or dromedaries. In all infected animals, the infection rate increased with the age of the host. CE cysts were predominantly found in both the liver and lung of the same animal in sheep and cattle. Aborted lesions were the most common stage of infection, and multiple CE cysts were frequently observed in affected animals. Fertile CE cysts were highly prevalent in both sheep and cattle, with rates increasing with host age, confirming the critical role of sheep in the parasite transmission cycle and demonstrating that cattle in Tunisia also play a significant role in the propagation of CE. Molecular analysis confirmed the predominance of the zoonotic G1 genotype of *E. granulosus* sensu stricto. This is particularly concerning as the G1 genotype is also the most common genotype affecting humans. This underscores a strong zoonotic potential and highlights the need for integrated control strategies. The findings emphasize the role of the livestock-dog cycle in CE transmission, posing risks to humans living near infected animals. Effective measures, including slaughter regulations, dog deworming, public education, and enhanced veterinary surveillance within a One Health approach, are essential for reducing CE’s impact on human and animal health.

## 1 Introduction

Cystic echinococcosis (CE), also known as hydatidosis, is a cosmopolitan zoonotic disease caused by the larval stage of the *Echinococcus granulosus sensu lato* (s.l.) species complex. CE has a worldwide distribution, with a high prevalence in North African countries, especially Tunisia (1–3). This disease is prevalent among humans and animals, particularly in rural areas where close interactions between livestock and dogs facilitate its transmission cycle. Considering its considerably high morbidity rate, CE is among the priority neglected diseases of the World Health Organization which included it in its strategic roadmap for the year 2022 (4). The annual economic loss due to this disease is estimated at approximately USD 3 billion worldwide (5). In addition to its public health impact, CE imposes a significant economic burden on livestock production, including the condemnation of infected viscera, reductions in meat and milk quality and production, and decreased fecundity (6, 7).

The life-cycle of the parasite requires two hosts: canids as definitive hosts and a wide range of herbivorous or omnivorous animals as intermediate hosts. The adult forms are small taeniid worms that develop in the midgut of canids, especially dogs, which release eggs in their feces. After ingestion by an intermediate host, the viable oncosphere transforms into a CE cyst (larval stage). The most commonly involved organs are the liver and lungs but many other anatomical sites may also be affected (8). The importance of traditional livestock farming is responsible for CE transmission due to the frequent close interactions between production animals and dogs. Human infection is caused by accidental ingestion of eggs through contaminated vegetables, water, soil and fomites or by direct contact with infected dogs (9).

Five species are currently recognized as responsible for CE: *E. granulosus sensu stricto* (s.s) (G1 and G3 genotypes), *E. equinus* (G4 genotype), *E. ortleppi* (G5 genotype), *E. canadensis* (G6–G10 genotypes) *and E. felidis* (10). *E. granulosus s.s.* (especially the G1 genotype) is the most common species associated with CE in both animals and humans worldwide, particularly in Tunisia (3, 11).

With a mean annual surgical incidence (ASI) of CE of 12.7 per 100,000 inhabitants and an annual economic loss estimated at USD 10–19 million (12), Tunisia is one of the most endemic countries in the Mediterranean area (13). On the basis of the findings of the latest national survey on CE, the 24 governorates (Tunisian administrative units) were classified as: hyperendemic (ASI > 19), holoendemic (12.7 < ASI < 19), mesoendemic (6.3 < ASI < 12.7) and hypoendemic (ASI < 6.3) regions (14).

While numerous studies have explored human and animal contamination in various parts of the country (3, 15–17), recent data on the prevalence of CE in livestock, especially in the mesoendemic areas of Tunisia, remain scarce.

This study aimed to assess the prevalence of CE in slaughtered animals, analyze the distribution of CE cysts within livestock, explore possible differences related to host species, and identify potential risk factors contributing to CE transmission dynamics which could target control strategies and enhance public health measures.

## 2 Materials and methods

### 2.1 Sample collection

**Figure 1:**
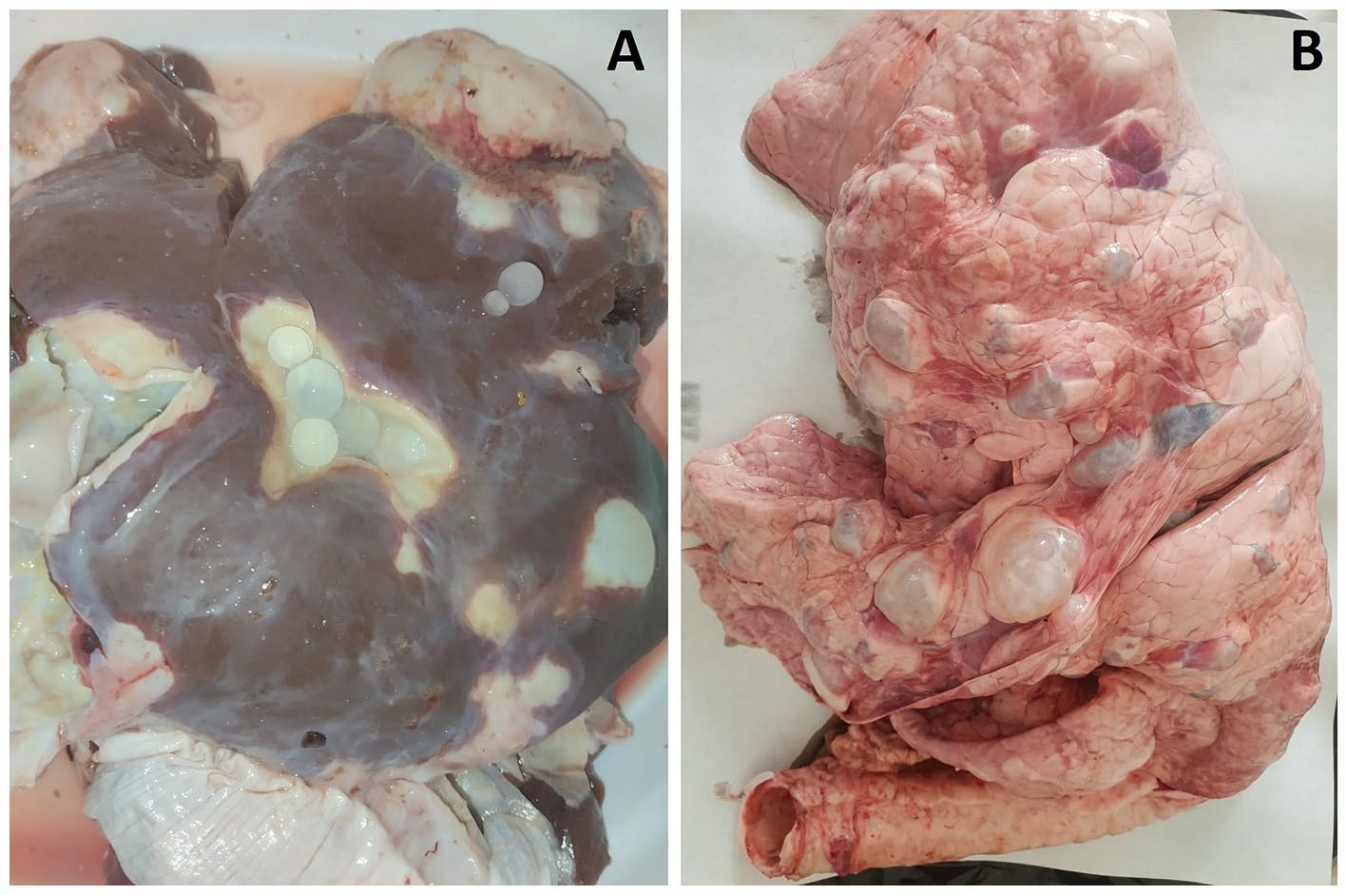
Multiple CE cysts observed in the liver **(A)** and lungs **(B)** of a sheep.

The study was conducted in the Sousse and Mahdia governorates (CE mesoendemic areas) located in the northeastern region of Tunisia. Owing to low rainfall, livestock husbandry in this area is primarily extensive and conducted on open rangelands. Livestock is widespread throughout the region, particularly in rural areas, with approximately 470,000 sheep female units (FUs), 33,000 goat FUs, 34,000 cattle FUs, and 586 camel FUs recorded (18). From January 2023 to August 2024, a total of 21,487 animals (12,847 sheep, 4,027 goats, 4,542 cattle and 71 dromedaries), slaughtered at the central abattoir of Sousse (governorate of Sousse; 35°47′58.45″N, 10°37′41.81″E) and the regional abattoir of Ksour Essef (governorate of Mahdia; 35°24′39.83″N, 10°59′35.64″E), were examined for the presence of *E. granulosus* s.l. cysts (Figure 1). To minimize potential sampling biases, multiple weekly visits were conducted throughout the study period. During each visit, all slaughtered animals were analyzed without any preselection.

**Figure 2:**
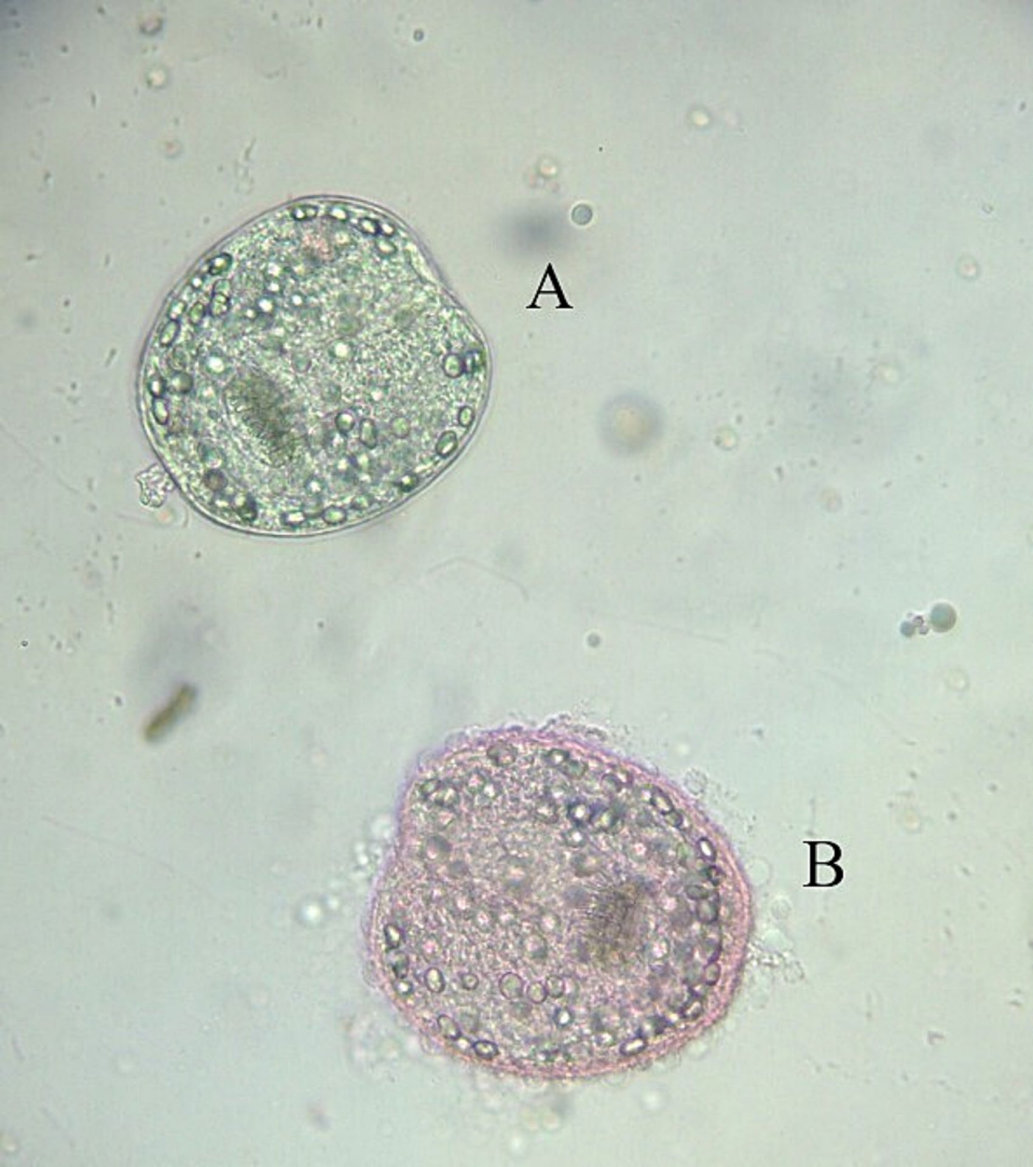
Assessment of protoscolex viability using 0.2% eosin staining. Alive **(A)** and dead **(B)** protoscolex.

CE cysts were identified during postmortem inspection through visual assessment, palpation, and systematic incision of the visceral organs. Animals with CE cysts in the liver, lung or both, were listed on a form assigned a unique identification number. For each contaminated animal, sex, age, and the number and localization of CE cysts were recorded. According to the international consensus on terminology, the lesional aspects of the CE cysts were classified as active, aborted (calcified), or caseous (purulent-like lesion) (19, 20). All CE cysts observed in slaughtered animals were recorded; however, only 480 active CE cysts from 118 sheep and 39 cattle were analyzed for fertility and protoscolex viability. CE cysts were collected from various animals and organs (liver and lung), with 1–2 CE cysts sampled per animal. When both organs were affected (liver-lung complex), CE cysts were collected from each site. CE cyst fertility (presence or absence of protoscoleces) was determined through light microscopic observation. Protoscolex viability was assessed using a 0.2% eosin vital stain, where viable protoscoleces remained unstained and non-viable ones were stained red (Figure 2). For each CE cyst, the percentage viability was calculated by counting the number of live protoscoleces per 100 observed protoscoleces. Due to budgetary constraints, a subset of 110 fertile CE cysts was selected for molecular genotyping: 81 from sheep (56 from the liver and 25 from the lungs) and 29 from cattle (23 from the liver and 6 from the lungs).

### 2.2 DNA extraction and PCR amplification

Protoscolex DNA was extracted using the phenol/chloroform method as described by Green and Sambrook (21). DNA amplification was performed via the Egss1 primer pair (5’GTATTTTGTAAAGTTGTTCTA3’ and 5’CTAAATCACATCATCTTACAAT3’), which specifically targets partial 12S rRNA mitochondrial gene of the G1 genotype of *E. granulosus s.s.* (22), the predominant genotype responsible for CE infections in slaughtered animals in Tunisia. Amplification was carried out in a final reaction volume of 50 μL containing 2 mM MgCl2, 200 μM of each dNTP, 40 pmol of each primer, 3 μL of DNA template, and 2.5 units of Taq (DreamTaq) in a 1× concentrated buffer, along with 3 μL of DNA template. The PCR program consisted of an initial denaturation step at 95°C for 5 min, followed by 40 cycles of denaturation (94°C for 30 s), annealing (57°C for 1 min) and extension (72°C for 40 s), with a final extension step at 72°C for 5 min. Positive and negative controls were included in each reaction. The PCR products were visualized through gel electrophoresis on a 1.5% agarose gel stained with a safe stain (Midori Green Advance DNA stain, Nippon Genetics EUROPE).

### 2.3 Statistical analysis

Statistical analyses were performed using SPSS software version 27. Qualitative data were expressed as percentages and compared using the chi-square (*χ*^2^) test for correlations. Odds ratios (OR) were calculated for 2×2 contingency tables to measure the strength of association between variables and assess relative risk. The quantitative data were expressed as means ± standard deviations. Depending on the nature of the data, the Mann–Whitney U test was used for nonparametric comparisons, and one-way ANOVA was applied for comparisons between multiple groups. The *Z*-test for two proportions and confidence interval (95% CI) were employed to compare specific proportions. Statistical significance was set at *p* < 0.05.

## 3 Results

### 3.1 Distribution of CE in slaughtered animals

During postmortem examination, 21,487 domestic ruminants were inspected for cystic echinococcosis. Among these, 2,038 sheep and 435 cattle harbored one or more CE cysts in liver and/or lungs. The average CE prevalence was 11.5% (95% CI [11.09–11.94]) and differed significantly between infected animals (*χ*2 = 46.626, *p* < 0.001). It was more common in sheep (15.86, 95% CI [15.25–16.51]) than in cattle (9.57, 95% CI [8.74–10.47]), whereas no cases were detected in goats (95% CI [0.004–0.9]) and dromedaries (95% CI [0.00–5.00]) (*Z* = 10.46941, *p* < 0.001). Among the 14,382 CE cysts observed in slaughtered animals, 11,344 were found in sheep and 3,038 in cattle (*Z* = 96.676, *p* < 0.001) (Table 1).

**Table 1:** CE prevalence in slaughtered animals.

	Number of examined animals	Number of infected animals (%)	Chi-square, *p*-value	Number of observed CE cysts (%)	Chi-square, *p*-value
Sheep	12,847	2,038 (15.86) a	*χ*2 = 46.626 *p* < 0.001	11,344 (78.87) a	*χ*2 = 10945.346, *p* < 0.001
Cattle	4,542	435 (9.57) b		3,038 (21.12) b	
Goats	4,027	0 (0) c		0 (0) c	
Dromedary	71	0 (0) d		0 (0) d	
Total	21,487	2,473 (11.50)		14,382 (100)	

Letters (a, b, c, d) indicate statistically significant differences between proportions in the respective row (*Z*-test, *p* < 0.05). Groups within each item sharing different letters are significantly different.

Chi^2^ *p*-value was set to 0.05.

Three types of CE cyst localizations were observed in infected animals, depending on the affected organ: lesions involving both the liver and lungs and lesions localized exclusively to either the liver or the lungs (Table 2). The liver-lung complex was the most frequent site of infection in 58.63% (95% CI [56.46–60.78]) of sheep and 54.02% (95% CI [49.21–58.78]) of cattle (*p* < 0.001). Liver localization ranked second, affecting 29.53% (95% CI [27.56–31.57]) of sheep and 33.56% (95% CI [29.14–38.22]) of cattle (*p* < 0.001), while pulmonary localization was the least common, observed in 241 sheep (11.82, 95% CI [10.45–13.31]) and 54 cattle (12.41, 95% CI [9.46–15.88]) (*p* < 0.001) (Table 2).

**Table 2:** Distribution of observed CE cysts based on organ localization, lesional aspect, and number of CE cysts in infected animals.

		Sheep		Cattle		Chi-square, *p*-value
		Number	%	Number	%	
Organ	Lungs	241	11.82% a	54	12.41% a	*χ*2 = 3.35, *p* = 0.187
	Liver	602	29.53% b	146	33.56% b	
	Liver-lung complex	1,195	58.63% c	235	54.02% c	
Lesional aspects of observed CE cysts *N* = 14,382	Active	1,210	10.66% a	453	14.91% a	*χ*2 = 45.023, *p* < 0.001
	Caseous	3,361	29.62% b	903	29.72% b	
	Aborted	6,773	59.70% c	1,682	55.36% c	
Number of CE cysts per organ in animals	1	288	14.13% a	62	14.25% a	*χ*2 **=** 0.576, *p* = 0.749
	2–10	1,231	60.40% b	255	58.62% b	
	>10	519	25.46% c	118	27.12% c	

Letters (a, b, c) indicate statistically significant differences between proportions in the respective columns (*Z*-test, *p* < 0.05).

Groups within each item sharing different letters are significantly different. Chi-2 significance was set at < 0.05.

Our study revealed that aborted CE cysts were the most common stage of development, representing 59.70% (95% CI [58.80–60.61]) of the CE cysts in sheep and 55.36% (95% CI [53.58–57.14]) in cattle. Caseous CE cysts accounted for approximately 30% of the CE cysts in both sheep and cattle, whereas the active stage was less frequent, with 1,210 CE cysts (10.66, 95% CI [10.10–11.25]) in sheep and 453 CE cysts (14.91, 95% CI [13.66–16.23]) in cattle. These differences were statistically significant (*χ*2 = 45.023, *p* < 0.001) (Table 2).

CE cyst lesional aspects in infected organs were comparable between sheep and cattle (Table 3) with aborted CE cysts predominating in the liver, with prevalence rates of 66.67% (95% CI [65.54–67.80]) in sheep and 67.53% (95% CI [65.25–69.77]) in cattle. In contrast, caseous CE cysts were significantly more prevalent in the lungs, with rates of 88.84% (95% CI [87.73–89.89]) in sheep and 88.37% (95% CI [86.10–90.39]) in cattle (*p* < 0.001).

**Table 3:** Fertility and lesional aspect of CE cysts, viability of protoscolex, and number of cysts depending on the localization of CE cysts.

		Sheep			Cattle		
		Liver	Lungs	Chi-square, *p*-value	Liver	Lungs	Chi-square, *p*-value
Number of fertile CE cysts *n* = 459		279 **a** (72.84%)	104 **b** (27.15%)	–	70 **a** (92.10%)	6 **b** (8.57%)	*χ*2 = 12.908, *p* < 0.001
Protoscolex viability		68,57 ± 24,21%	66,10 ± 22,75%	0.268*	63.23 ± 25.38%	64.25 ± 23.10%	0.742*
Number of CE cysts per organ in animal	1	160 a (55.55%)	128 a (44.44%)	*χ*2 = 18.408, *p* < 0.001	34 a (54.83%)	28 a (45.16%)	*χ*2 = 5.10327, *p* = 0.07795
	2–10	554 b (45%)	677 b (55%)		110 b (43.13%)	145 b (56.86%)	
	>10	207c (39.88%)	312 c (60.11%)		44 c (37.28%)	74 c (62.71%)	
Lesional aspects of observed CE cysts *N* = 14,382	Active	760 **a** (62.80%)	450 **b** (37.19%)	*χ*2 = 2856.627, *p* < 0.001	279 **a** (61.58%)	174 **b** (38.41%)	*χ*2 = 763.129, *p* < 0.001
	Caseous	375 **a** (11.15%)	2,986 **b** (88.84%)		105 **a** (11.62%)	798 **b** (88.37%)	
	Aborted	4,516 **a** (66.67%)	2,257 **b** (33.32%)		1,136 **a** (67.53%)	546 **b** (32.46%)	

*n = Total number of analyzed CE cysts; N = Total number of observed CE cysts.*

Letters (a, b) in bold indicate statistically significant differences between proportions in the respective row (*Z*-test, *p* < 0.05). Groups within each animal sharing diffrent letters are significantly different.

Letters (a, b, c) indicate statistically significant differences between proportions in the respective column (*Z*-test, *p* < 0.05). Groups within each organ sharing different letter are significantly different.

Chi-2 significance was set at < 0.05. *p-value of Mann–Whitney Test U.

Among the 14,382 CE cysts observed in host animals, 480 active CE cysts (280 from sheep livers, 109 from sheep lungs, 79 from cattle livers, and 12 from cattle lungs) were collected during the study, of which 459 (95.62, 95% CI [93.39–97.27]) were identified as fertile and 21 (4.37, 95% CI [2.73–6.61]) as sterile (Table 3). In sheep, a greater percentage of fertile CE cysts was found, with 383 (98.45, 95% CI [96.67–99.43]) fertile CE cysts and only 6 (1.54, 95% CI [0.57–3.33]) sterile CE cysts, with a protoscolex viability rate of 67.80 ± 23.63%. In contrast, 76 (86.81, 95% CI [74.27–90.47]) fertile CE cysts and 15 (16.48%, 95% CI [9.53–25.73]) sterile CE cysts were identified in cattle (χ2 = 12.908, *p* < 0.001) (Table 3), with a protoscolex viability rate of 52.80 ± 24.15%. For both ovine and bovine samples, the prevalence of fertile CE cysts in the liver was significantly higher than that in the lungs. Specifically, sheep exhibited 279 fertile hepatic CE cysts (72.84%) compared to 104 fertile pulmonary CE cysts (27.15%) (*Z* = 12.646, *p* < 0.001) (Table 3). Cattle presented an even more pronounced hepatic predilection, with 92.10% of fertile CE cysts (*n* = 70) found in the liver and only 8.57% (*n* = 6) in the lungs (*Z* = 10.3822, *p* < 0.001).

Protoscolex viability within fertile CE cysts was consistent between lung and liver localization in each infected animal, with no significant differences observed between host species (Table 3). The overall protoscolex viability rate in sheep was 68.57 ± 24.21% for liver CE cysts and 66.10 ± 22.75% for lung CE cysts, whereas in cattle, it was 63.23 ± 25.38% for liver CE cysts and 64.25 ± 23.10% for lung CE cysts.

The distribution of the number of CE cysts per organ was similar between sheep and cattle (*χ*2 = 0.576, *p* = 0.749). The majority of the animals harbored between 2 and 10 CE cysts, with 60.40% (*p* < 0.001) being sheep and 58.62% being cattle (Table 2). Animals with more than 10 CE cysts accounted for 25.46% of the sheep and 27.12% of the cattle, while those with only one CE cyst were the least common, representing 14.13% of the sheep and 14.25% of the cattle.

### 3.2 Fertility of CE cysts and protoscolex viability according to the sex and age of slaughtered animals

**Figure 3:**
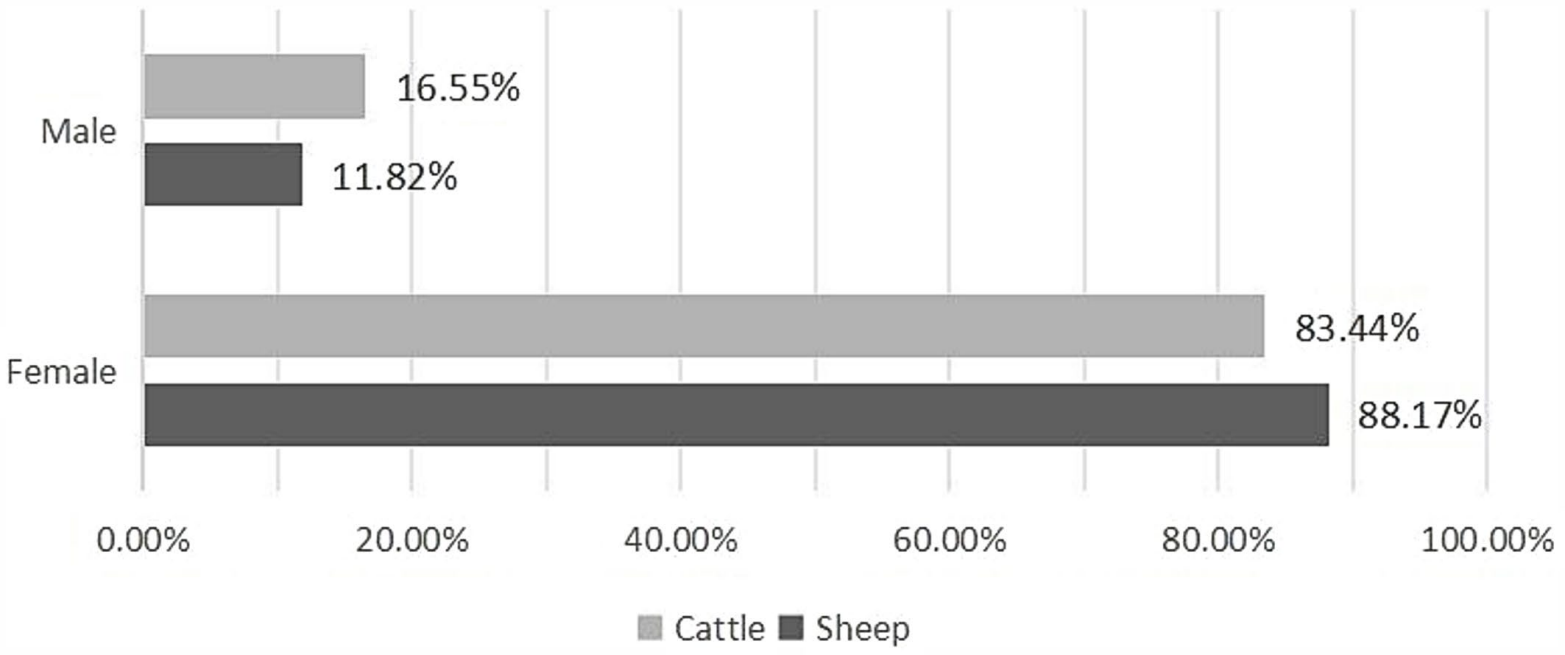
Sex-based prevalence of CE in infected animals.

Regarding all slaughtered animals, the results highlighted that in both sheep (OR = 24.324 [21.112–28.025]) and cattle (OR = 34.473, IC 95% [26.343–45.112]), CE was more commonly observed in females than in males (Figure 3; Table 4).

**Table 4:** Influence of the sex of infected animals on the fertility of CE cysts and the viability of protoscolices.

	Sheep				Cattle				OR, OR 95% CI	*p*-value
	Male	Female	OR, [OR 95% CI]	*p*-value	Male	Female	OR, [OR 95% CI]	*p*-value		
Number of examined animals	8,514	4,333	24.324 [21.112–28.025]*	*p* < 0.001	3,655	887	34.473 [26.343–45.112]*	*p* < 0.001	1.780 [1.595–1.986]**	*p* < 0.001
Number of infected animals	24 a (11.82%)	1,797 b (88.17%)			72 a (16.55%)	363 b (83.44%)				
Number of fertile CE cysts *N* = 459	184 a (48.40%)	199 b (51.95%)	0.083 [0.004–1.486]*	*p* < 0.001	6 a (7.89%)	70 b (92.10%)	2.858 [0.1528–53.450]*	*p* = 0.03	12.598 [4.736–33.509] **	*p* < 0.001
Number of sterile CE cysts *N* = 21	0 a (0%)	6 b (100%)			0 a (0%)	15 b (100%)				
Protoscolex viability	81.25 ± 16.67%	64.90 ± 23.90%	–	*p* < 0.001	50.00 ± 00.00%	53.00 ± 24.99%	–	*p* = 0.883	–	*p* < 0.001

*N = total number of analyzed CE cysts.*

Letters (a, b, c) indicate statistically significant differences between proportions in the respective row (*Z*-test, *p* < 0.05). Gender groups within each item sharing the same letter are not significantly different. *The exposed groupe is Females; **The exposed groupe is sheep.

The collection of fertile CE cysts from sheep was comparable across both sexes, whereas in cattle, a significantly higher proportion of fertile CE cysts (92.10%) was found in females compared to males (*p* < 0.001) (Table 4).

Protoscolex viability within fertile CE cysts was comparable between male and female cattle. However, in sheep, a significant difference was observed, with males exhibiting a higher viability rate (81.25 ± 16.67%) than females did (64.90 ± 23.90%). Sterile CE cysts were exclusively observed in females of both host species (Table 4).

**Figure 4:**
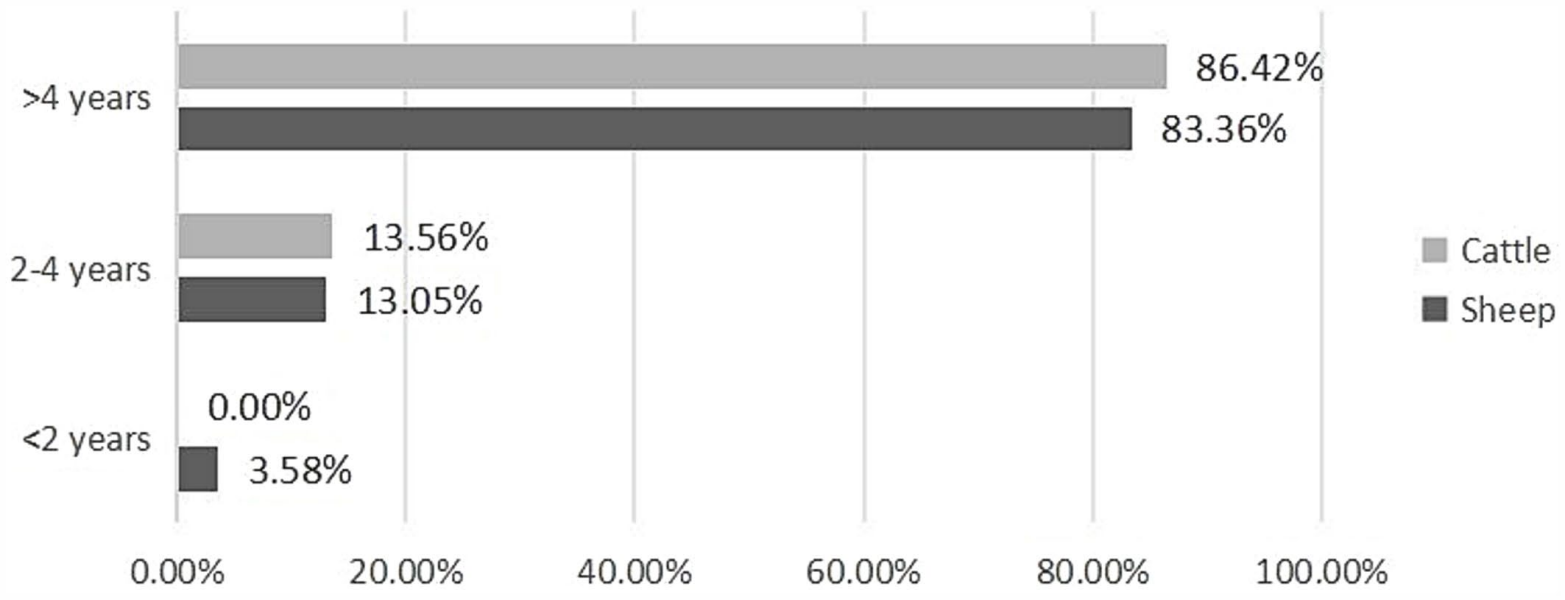
Age-based prevalence of CE in infected animals.

The infection rate increased significantly with the age of the host across all studied slaughtered livestock (*Z* = 12.152, *p* < 0.001) (Figure 4; Table 5). Our findings revealed that the prevalence of CE was highest in animals older than 4 years, with 83.36% (95% CI [81.68–84.96]) in sheep and 86.42% (95% CI [82.86—89.51]) in cattle, whereas the lowest prevalence was observed in animals younger than 2 years, with 3.58% (95% CI [2.82–4.48]) in sheep and 0% (95% CI [0.00–8.00]) in cattle (Table 5).

**Table 5:** Influence of the age of infected animals on the fertility of CE cysts and the viability of protoscolices.

	Sheep				Cattle			
	<2 years	2–4 years	>4 years	Chi-square, *p*-value	<2 years	2–4 years	>4 years	Chi-square, *p*-value
Number of examined animals	7,066	1,668	4,113	*χ*2 = 2157.551, *p* < 0.001	1,708	1,937	897	*χ*2 = 937.546, *p* < 0.001
Number of infected animals	73a (3.58%)	266b (13.05%)	1699c (83.36%)		0a (0%)	59b (13.56%)	376c (86.42%)	
Number of fertile CE cysts *N* = 459	24a (6.26%)	63b (16.44%)	296c (77.28%)	*χ*2 = 1.755, *p* = 0.415	0a (0%)	0a (0%)	76b (100%)	NA
Number of sterile CE cysts *N* = 21	0a (0%)	0a (0%)	6b (100%)		0a (0%)	0a (0%)	15b (100%)	
Protoscolex viability	78.57 ± 10.69%	61.79 ± 26.05%	76.11 ± 16.40%	*p* < 0.001*	0.00 ± 0.00	0.00 ± 0.00%	52.80 ± 24.15%	NA

*N = total number of analysed CE cysts; NA: not applicable.*

Letters (a, b, c) indicate statistically significant differences between proportions in the respective row (*Z*-test, *p* < 0.05). Age groups within each item sharing different letters are significantly different. Chi-2 significance was set at < 0.05. *Anova one way test *p*-value.

In cattle, all the fertile CE cysts were collected from animals older than 4 years, with a protoscolex viability rate of 52.80 ± 24.15%. Similarly, 76.11 ± 16.40% of fertile CE cysts containing viable protoscoleces in sheep were found in animals older than 4 years. The prevalence of fertile CE cysts in sheep was 6.26% in animals younger than 2 years and 16.44% in those aged between 2 and 4 years. Sterile CE cysts were exclusively observed in sheep and cattle aged over 4 years (Table 5).

### 3.3 PCR-based molecular evaluation of CE cysts

Out of a total of 459 fertile CE cysts collected, 81 from sheep and 29 from cattle were molecularly genotyped. All the CE cysts were successfully amplified targeting a 254 bp fragment of the partial 12S rRNA mitochondrial gene and were identified as belonging to the G1 genotype of *E. granulosus* sensu stricto. This genotype was observed regardless of CE cyst localization (liver or lungs) and the type of slaughtered animal (sheep or cattle).

## 4 Discussion

The prevalence of CE in the slaughtered animals examined in this study highlights significant economic and health challenges in the livestock sector. Specifically, CE-related economic losses in Tunisia have been estimated at US$ 14.7 million, including costs associated with the condemnation of infected organs (US$ 1.76 million), reduced meat and milk production (US$ 16.1 million), decreased fertility in infected animals (US$ 4.02 million), and losses due to reduced livestock productivity in wool (US$ 1.18 million) (12). However, the impact of CE infection varies across host species (ovine, bovine, goat or dromedary). In our study, sheep presented a higher prevalence rate (15.86%) compared to cattle (9.57%), which aligns with the findings of Lahmar et al. (15) in Tunisia, who reported a prevalence of 16.42% (95% CI [15.05–17.87%]) in sheep and 8.56% (95% CI [7.68–9.46%]) in cattle. Comparable differences in CE infection rates have been reported in other Mediterranean countries, such as Italy, Greece, and Algeria, where sheep display higher infection rates than cattle do (23, 24). The elevated prevalence in sheep is largely attributed to their grazing habits in open pastures where they are exposed to *E. granulosus* s.l. eggs shed by infected dogs. Several studies conducted in Tunisia have highlighted significant environmental contamination with parasite eggs (16, 25, 26). Prolonged exposure increases the likelihood of ingesting parasite eggs, thereby resulting in a higher incidence of CE cyst development. In contrast, cattle, which are typically raised in more controlled environments and fed industrial concentrates, have reduced contact with contaminated environments, explaining their lower infection rate. On the other hand, no cases of CE were observed in dromedaries and goats during this study. These findings are similar to those reported in Tunisia, where CE prevalence in goats ranged from 1.7 to 2.88% and from 0.8 to 5.94% in dromedaries (15, 16). They also align with global studies conducted in Yemen (27), Iran (28), Algeria (29), and China (30). These prevalences are likely attributable to the distinct feeding behaviors of these species as both primarily consume elevated vegetation, such as shrubs and bushes, thereby minimizing their exposure to *E. granulosus* s.l. eggs. This trend may also be explained by the fact that these animals are often slaughtered at a young age, before the parasite has sufficient time to develop into CE cysts, a process that typically requires several months (31).

A clear correlation was observed between animal age and the CE prevalence, with older animals being significantly more affected. Animals over 4 years of age exhibited the highest infection rates, reaching 83.36% in sheep and 86.42% in cattle. This age-related trend is consistent with the findings of numerous studies, where prolonged exposure to *E. granulosus* s.l. eggs increases the risk of contamination. Furthermore, an animal’s extended lifespan provides more time for CE cysts to develop and mature within the host (15, 19, 32, 33, 56). In contrast, younger animals, especially those under 2 years of age, show markedly lower prevalence, as they are generally slaughtered before the larvae complete their developmental cycle. Gender also plays a significant role in CE prevalence, with females showing higher infection rates than males. This disparity can be explained by livestock management practices, where females are kept longer for breeding and milk production, unlike males who are typically slaughtered at a younger age, leading to a greater cumulative exposure to the parasite over time.

The liver-lung complex was the most common site of CE cyst localization in both sheep and cattle, followed by CE cysts confined to either the liver or lungs alone. This distribution reflects the known migration pathway of *E. granulosus* embryos, which first pass through the liver as the primary filter and then through the lungs as the secondary filter (32, 34). The frequent occurrence of hepatic CE cysts is explained by the liver’s proximity to the digestive tract, with larvae migrating through the portal vein as part of their developmental route. However, in some cases, larvae bypass the liver entirely, traveling through the vena cava system to the lungs, where they develop into pulmonary CE cysts (35).

The presence of multiple CE cysts in infected animals is attributed to widespread environmental contamination with *E. granulosus* eggs, resulting in high levels of exposure (16, 25, 26). Furthermore, a study conducted by M’rad et al. (36), which assessed the genetic diversity of CE cysts using microsatellites, demonstrated successive infection events in host species (ovine and bovine) with *E. granulosus* eggs. The majority of sheep and cattle harbored between 2 and 10 CE cysts, whereas a smaller percentage of animals carried more than 10 CE cysts. These findings corroborate a study conducted in Sardinia, which demonstrated that most infected animals develop fewer than 10 CE cysts (37). The development of multiple CE cysts may be linked to the host’s immune response, which, despite its efforts to control the infection, allows the parasite to persist through various immune evasion strategies (38). Moreover, the high proportion of aborted CE cysts observed in the present study, especially in older animals, and also reported in Mauritania and Italy (19), indicates a chronic phase of infection. Calcification is part of the immune system’s efforts to contain the parasite, limit CE cyst viability, and reduce the risk of transmission, but does not eliminate the parasite entirely from the host (39).

The fertility of CE cysts plays a crucial role in sustaining the *E. granulosus* s.l. life cycle, as fertile CE cysts containing viable protoscoleces can develop into adult parasites in definitive hosts. In this study, a high fertility rate was observed in both sheep (98.45%) and cattle (86.81%), with 52.80 ± 24.15% to 67.60 ± 23.63% of the protoscoleces being viable. This high fertility rate was particularly notable in sheep, with no significant difference between sexes, suggesting that both male and female sheep contribute equally to the transmission cycle. In cattle, a striking difference was observed between sexes, with 92.10% of fertile CE cysts found in female cattle, while oxen showed a much lower prevalence of fertile CE cysts. This disparity may be attributed to the longer lifespan of females due to their reproductive role. The prevalence of fertile CE cysts in older animals demonstrates that prolonged longevity correlates with an increased likelihood of developing fertile CE cysts capable of infecting definitive hosts (40). The high fertility and viability rates observed in older breeding females underscore the importance of targeting high-risk animals in control strategies to reduce transmission risks, particularly by ensuring the proper disposal of contaminated offal. Furthermore, the high fertility observed in cattle is characteristic primarily of North African countries (41, 42) and was also noted in the present study. Indeed, previous studies conducted worldwide indicate that *E. granulosus s.s.* (G1 genotype) cysts, especially in cattle, tend to exhibit low fertility rates (35). National surveillance conducted at French slaughterhouses revealed that CE cysts in cattle are often sterile, with only a minority containing viable protoscoleces (43). This low fertility, with a viability of approximately 5% in some studies, is notably lower than that in other intermediate hosts such as sheep (9). In contrast, in Tunisia, cattle appear to play an active role in maintaining the parasite’s life cycle, posing a risk for dog contamination and, consequently, for human and animal infection. This finding reinforces the importance of targeting cattle in the implementation of control strategies. This observed difference in fertility could be influenced by the regional haplotype of *E. granulosus* s.s., potentially exhibiting greater compatibility with cattle in North African environments (32, 44–47). For both ovine and bovine hosts, the percentage of fertile CE cysts in the liver was significantly higher (ranging from 72.84% in sheep to 92.10% in cattle) than that in the lungs, which is comparable with the situation in Libya and Iraq (reaching 53.6% in the liver and 14.29% in the lung) (48, 49). However, this variation in fertility between hepatic and pulmonary CE cysts has been shown to differ across studies (50–52). The reasons for these differences remain unclear but may be attributed to several factors, including the developmental stage of the CE cyst, the host’s immune response, and the specific physiological conditions of each organ.

In Tunisia, CE infestation in livestock is due primarily to the G1 genotype of *E. granulosus s.s.* (16, 42, 44). This is comparable to the epidemiological situation across Mediterranean countries, such as Algeria (29), Italy (19), and Greece (53), emphasizing its significant role in both local and global transmission dynamics. In Tunisian livestock, the high prevalence of this genotype raises concerns not only for its economic impact on animal productivity and veterinary health but also for its implications for public health. Indeed, the G1 genotype of *E. granulosus* s.s. is, in fact, the primary genotype responsible for human CE cases (54), underlining the zoonotic risk posed by insufficient control of transmission pathways. Phylogeographic analyses, conducted by Kinkar et al. (46), highlight the significant transmission route originating from countries such as Tunisia, which, along with Turkey and Argentina, served as a pathway for G1 genotype dispersion globally. This spread is largely driven by human activities, such as the livestock trade, rather than the natural migration of definitive hosts (46). Factors such as unregulated home slaughter practices, stray dog density, lack of awareness about the CE transmission cycle, and the feeding of raw offal to dogs, facilitate the parasite’s life cycle (2, 24, 25). The World Health Organization recognizes the control and prevention of CE as critical priorities, particularly within a One Health approach, owing to its significant impact on human and animal health, as well as on food supply chains. The practical application of the “One Health” approach involves establishing integrated surveillance networks to monitor CE prevalence in both humans and animals, focusing on identifying high-risk zones, tracking transmission patterns, and evaluating the effectiveness of control measures. Regular community-based deworming programs targeting definitive hosts, such as stray and domestic dogs, are a priority, particularly in high-prevalence areas. These programs should include the provision of cost-effective anthelmintics, community incentives to encourage participation, and collaboration with local authorities and veterinary services to ensure sustainability. Additionally, stricter enforcement of existing slaughterhouse regulations aimed at ensuring the proper disposal of infected offal, particularly from older animals, is crucial for reducing environmental contamination caused by infected dog feces. Public education campaigns must be launched to raise awareness among rural communities about the life cycle of *Echinococcus granulosus* s.l., the importance of hygiene, and the risks of feeding dogs raw offal. This behavior highlights the significant role that humans play in maintaining the parasite’s life cycle, as feeding dogs raw viscera containing viable CE cysts contributes to environmental contamination with *E. granulosus* eggs and transmission to livestock. Leveraging local communication channels and involving community leaders can enhance the impact of these campaigns. Furthermore, fostering interdisciplinary collaboration among veterinarians, medical professionals, policymakers, and environmental experts is key to addressing the socioeconomic and ecological factors influencing CE transmission. Potential barriers to implementing the proposed strategies include high densities of stray dogs, which complicate efforts to control definitive hosts through deworming programs. The limited resources for capturing and managing stray dogs exacerbate this issue. Many proposed interventions, such as integrated surveillance systems, regular deworming programs, and enhanced slaughterhouse practices, require significant funding and logistical coordination, which may be challenging in resource-limited settings.

While this study provides valuable insights into the epidemiology of cystic echinococcosis in central Tunisia, several limitations must be acknowledged to contextualize the findings and guide future research efforts. The study is restricted to two central slaughterhouses that process animals from various parts of the country. Although this allows for some extrapolation of the national epidemiological situation, the findings primarily reflect mesoendemic areas in central Tunisia. Expanding the study to include slaughterhouses from other regions with varying endemicity levels would further enhance our knowledge of the spatial distribution of the disease. Another important limitation is that only a subset of the collected cysts was molecularly genotyped, rather than all observed and isolated CE cysts. As a result, we cannot rule out the possibility that additional genotypes were present but not detected in this study. This highlights the importance of expanding genotyping efforts in future studies to further refine our understanding of the genetic diversity of *E. granulosus* s.l. in Tunisia. Additionally, conducting phylogenetic analyses with another marker, such as the Nad5 gene (55), could improve our understanding of the relationships among the CE cysts sampled in this study and those from different regions. This comparative approach would provide information about the evolutionary dynamics and genetic diversity of the pathogen, offering a more comprehensive view of CE epidemiology in Tunisia and beyond.

## 5 Conclusion

In conclusion, this study underscores the significant zoonotic implications of CE in Tunisia, where the G1 genotype of *E. granulosus* s.s. is prevalent across livestock. The high infection rates observed, particularly in sheep, point to substantial public health risks and ongoing transmission, influenced by local livestock management practices and environmental exposure. The associations between infection rates and factors such as host species, age, and management practices highlight the need for targeted control measures. Addressing CE in Tunisia requires integrated, multisectoral strategies to limit transmission, with a focus on stricter enforcement of existing slaughterhouse regulations, increased public awareness, and targeted deworming programs for definitive hosts, particularly in high-prevalence zones. Further research on the genetic diversity and local adaptations of the G1 genotype will be essential to refine control strategies and reduce the CE burden on both animal health and public health systems. These strategies, combined with enhanced veterinary surveillance, can significantly contribute to breaking the life cycle of the parasite and reducing its burden on both human and animal populations.

## Data Availability

The original contributions presented in the study are included in the article further inquiries can be directed to the corresponding author.
